# Microbial Musings – July 2020

**DOI:** 10.1099/mic.0.000965

**Published:** 2020-07-30

**Authors:** Gavin H. Thomas

**Affiliations:** ^1^​ Department of Biology, University of York, York, UK

We start this month with an inspiring Personal View from six early-career researchers who dropped everything and moved to Milton Keynes [1] . For those of you not familiar with UK topography, this is certainly not an everyday occurrence, although nothing about the last 5 months has been ordinary. These six young microbiologists and life scientists, thrown together to form one of the teams in the UK Lighthouse Labs set up as a response to an urgent need to increase coronavirus disease 2019 (COVID-19) testing capacity, recount what it was like to be involved in this epic effort. The team comprising Fatima Ulhuq (@Fatimaaa_U), Sophia Berry (@DrSophiaBerry), Lucy Kelly (@micro_lucy), Ben Stansfield (@StansfieldBen), Anna Deal (@Annacadeal) and Harriet Lester, emphasize the strong feeling of purpose and community that was quickly created, knowing that they were helping a national effort. This was particularly poignant for Harriet, who had lost her mother to the disease shortly after she had agreed to join the project. It is a gripping read and I highly recommend it to you. Two other young microbiologists have also written about their experiences, Ellie Boardman (@ellierboardman) for the Microbiology Society and Adrienne Adele Cox (@AdrienneACox) for The Biologist. These young scientists and many of their colleagues deserve our recognition and thanks for their sterling work and have likely forged friendships that will last for decades.

We start this month’s science with a very interesting review article on isoprene. This is a molecule that I really had very little awareness of, but it is a major volatile hydrocarbon emitted in vast quantities by trees and other vegetation. The authors of the review, Colin Murrell and Andrew Crombie from the University of East Anglia, UK, and Terry McGenity (@TMcgenity), from the University of Essex, UK, point out that this chemical, more formally known as 2-methyl-1,3 butadiene, is produced in the biosphere in quantities of around 500 teragrams (Tg) of carbon per year [2], which is of a similar magnitude to that of methane! The chemical has complex interactions in the atmosphere, being implicated in both warming and cooling events. It seems natural, then, that microbes might have a role in the turnover of this compound, although it took until the late 1990s through the pioneering work of Cory Cleveland (@clevelac) and Joseph Yavitt from Cornell University, USA [3], to discover that soil microbes could remove isoprene from the atmosphere to almost undetectable levels. A strain of the actinobacterium *Rhodococcus* AD45 has been widely studied for its isoprene-degrading capabilities, with the full pathway being encoded on a large plasmid [4]. One of the outstanding questions in this field is how much the microbial turnover of isoprene contributes to the global cycling of this chemical. While the *iso* genes identified for the catabolic pathway in *Rhodococcus* are seen in related actinobacteria, there are also phenotypic data suggesting that a range of Gram negative bacteria can also degrade it using what appear to be unrelated routes, meaning that there is still lots more to learn about the role of bacteria in isoprene turnover.

This week I was fortunate enough to be able to present at my first proper international virtual conference, FASEB Microbial Glycobiology 2020 (@2020Faseb), sponsored by *Microbiology*. Although the science and organization were excellent, it was still a rather weird experience and the inability to chat to speakers and other attendees about their exciting science left me with a rather empty feeling, which I think just illustrates how much of a social activity science is. The cancellation of the Microbiology Society annual conference for 2021 is understandable and this early decision now gives the Society plenty of time to find creative alternatives, and we hope to create opportunities for authors in *Microbiology* to present their work in some way. The glycobiology conference started with a session on the gut microbiome chaired by Nathalie Juge (@JugeLab) of the Quadram Institute in Norwich, UK, and Nicole Koropatkin (@nkoropatkin) was one of many speakers to mention the gut commensal *Bacteroides thetaiotaomicron *, which is one of the few bacterial species names I have never quite known how to pronounce! Its name relates to a phenomenon explored in a paper in this issue about nutrient-dependent morphological variability [5]. As I have now learned from the introduction of this article, written by Aathmaja Rangarajan (@Aathmaja) and Julie Biteen (@JulieBiteenLab), with their University of Michigan, USA, colleague Koropatkin, the species name reflects an observation of its discoverer, Arcangelo Distaso, as down the microscope he could see the bug exhibiting morphologies that resembled the Greek letters theta, iota and omicron, which come together in the species name to form thetaiotaomicron. In this paper the authors chanced on the observation that when they grew *B. thetaiotaomicron* (*Bt*) in non-typical carbon-limited media they observed that the cells exhibited an extended rod morphology compared to the almost cocci shapes when grown with maltose as a good carbon source. This effect was also amplified by sodium bicarbonate, and the same effect could be replicated with *Escherichia coli* cells. While a mechanism for this is not elucidated, the authors point out that adaptive morphological variation through changes in growth media, and hence local environmental changes, could be important *in vivo*, especially for microbes that have evolved exclusively in complex communities with rapidly changing nutritional conditions.

Our next two papers switch to biofilms and the first is from the group of Cynthia Whitchurch (@Cwhitch), who is newly arrived in the UK after joining the Quadram Institute in 2019 after many years at the University of Technology, Sydney, Australia. The authors, who include first author Laura Nolan (@LauraNolanLab), who has also moved to the UK to take up a group leader position at Imperial College, Laura McCaughey (@LauraCMcCaughey) and Lynne Turnbull (@lynnet3), were studying the nutritional regulation of twitching motility in *Pseudomonas aeruginosa* biofilms. This process, by which the bacterium can swarm across an agar plate using type IV pili, is controlled by the Chp chemosensory apparatus in the cell membrane, which is similar to the Che system in *E. coli *. The authors were studying the function of ChpC, an orthologue of the CheW protein from *E. coli *. These proteins enable the chemosensory system to integrate additional signals and by using a *chpC* mutant strain the authors were able to observe that the enhancement of swarming that is seen with the host factors serum albumin and mucin is dependent on the function of ChpC. They suspected that the common link between these stimulating substances was that they could be broken down to oligopeptides and indeed when extracellular protease activity was inhibited the stimulation was lost. Individual amino acids were also unable to induce the stimulation, suggesting that oligopeptides were the important factor. What they now need to determine is how these oligopeptides are being sensed, as they could conceivably interact directly with the MCP, be bound by a substrate binding protein, or interact directly with ChpC.

Our second biofilm paper concerns an organism in which biofilms are much more poorly understood compared to *P. aeruginosa *. This is the bacterium *Mycoplasma pneumoniae *, an unusual cell wall-less pathogen with a very small genome. This can cause respiratory infections such as atypical pneumonia and tracheobronchitis, and can be treated with macrolide antibiotics. The paper from the group of Mitchell Balish at Miami University, USA, shows that these biofilms are very refractive to chemical dispersion and indicates that sonication is the only way to effectively break them up *in vivo*, while retaining cell viability [6]. It is not entirely clear why they are so hard to disperse and while they are known to secrete an exopolysaccharide [7], chemicals that should break this down did not lead to biofilm dispersion. Using their new dispersion method, they then compared the properties of the bacteria in early and late stage biofilms compared to dispersed biofilms. A number of key virulence factors are known for this microbe, including the ability to form reactive H_2_O_2_ and H_2_S [8] and a toxin [9]. These decreased in production as the biofilm matured. In contrast, the authors showed that the biofilms are much more resistant to macrolides and complement-mediated killing than the dispersed cells. They propose a model for the stages in biofilm formation and the typical properties of each stage, which should serve as a strong starting point for future work on how this pathogen behaves in the body.

Our final paper this month is on persisters, bacteria that are alive but have temporarily shut down metabolism to help them survive various stresses, such as antibiotics [10]. This study is on persister formation by the important human pathogen *Staphylococcus aureus *. While persisters have been shown to form readily from laboratory strains, in this paper from Hanne Ingmer (@HiIngmer) and colleagues at the University of Copenhagen, Denmark, the ability of clinical strains to develop this phenotype is examined [11]. The authors show that all the strains have the ability to form persisters, but that one particular collection of strains, clonal complex 30 (CC30), did this at 100 times higher frequency than the other isolates. The mechanism for this is unclear, as CC30 cells appear to have the same basal levels of ATP as the other clinical strains, and very similar levels of membrane potential, important factors that predispose cells to switch to persister status. CC30 strains do show a number of changes to typical virulence factors that are found in other clinical strains [12], which might explain this disposition for persister formation, but the mechanism awaits discovery.

Finally, this month I take over as Editor-in-Chief from Tanya Parish (@ProfTanya13), who has led the journal successfully for the last 5 years. I would like to thank Tanya for her wonderful leadership and dedication to the journal over many years. As I look at our amazing set of editors and senior editors I would also like to thank them and you as authors and readers and hope you can join me in ‘publishing for the community’ as we take the journal forward towards its 75th Anniversary in 2022.

## References

[1] Ulhuq FR, Berry SK, Kelly L, Stansfield B, Deal A (2020). Collaboration during a crisis - the Lighthouse Lab volunteers. Microbiology.

[2] Murrell JC, McGenity TJ, Crombie AT (2020). Microbial metabolism of isoprene: a much-neglected climate-active gas. Microbiology.

[3] Cleveland CC, Yavitt JB (1998). Microbial consumption of atmospheric isoprene in a temperate forest soil. Appl Environ Microbiol.

[4] Crombie AT, Khawand ME, Rhodius VA, Fengler KA, Miller MC (2015). Regulation of plasmid-encoded isoprene metabolism in Rhodococcus, a representative of an important link in the global isoprene cycle. Environ Microbiol.

[5] Rangarajan AA, Koropatkin NM, Biteen JS (2020). Nutrient-dependent morphological variability of *Bacteroides thetaiotaomicron*. Microbiology.

[6] Feng M, Schaff AC, Balish MF (2020). *Mycoplasma pneumoniae* biofilms grown *in vitro*: traits associated with persistence and cytotoxicity. Microbiology.

[7] Simmons WL, Daubenspeck JM, Osborne JD, Balish MF, Waites KB (2013). Type 1 and type 2 strains of Mycoplasma pneumoniae form different biofilms. Microbiology.

[8] Hames C, Halbedel S, Hoppert M, Frey J, Stülke J (2009). Glycerol metabolism is important for cytotoxicity of Mycoplasma pneumoniae. J Bacteriol.

[9] Kannan TR, Baseman JB (2006). Adp-Ribosylating and vacuolating cytotoxin of Mycoplasma pneumoniae represents unique virulence determinant among bacterial pathogens. Proc Natl Acad Sci U S A.

[10] Lewis K, cells P (2007). Dormancy and infectious disease. Nat Rev Microbiol.

[11] Liu L, Wang Y, Bojer MS, Andersen PS, Ingmer H (2020). High persister cell formation by clinical *Staphylococcus aureus* strains belonging to clonal complex 30. Microbiology.

[12] Cheung GYC, Kretschmer D, Duong AC, Yeh AJ, Ho TV (2014). Production of an attenuated phenol-soluble modulin variant unique to the MRSA clonal complex 30 increases severity of bloodstream infection. PLoS Pathog.

